# Floral attraction and flower visitors of a subcanopy, tropical rainforest tree, *Fontainea picrosperma*

**DOI:** 10.1002/ece3.7850

**Published:** 2021-07-07

**Authors:** Elektra L. Grant, Helen M. Wallace, Peter R. Brooks, Chris Burwell, Paul W. Reddell, Steven M. Ogbourne

**Affiliations:** ^1^ Genecology Research Centre University of the Sunshine Coast Sippy Downs Qld Australia; ^2^ Centre for Planetary Health and Food Security and Griffith School of Environment and Science Griffith University Nathan Qld Australia; ^3^ Biodiversity and Geosciences Program Queensland Museum South Brisbane Qld Australia; ^4^ Griffith School of Environment and Science Griffith University Nathan Qld Australia; ^5^ EcoBiotics Limited Yungaburra Qld Australia

**Keywords:** Australian Wet Tropics, dioecious, EBC‐46, Euphorbiaceae, floral scent, generalist pollination syndrome, tigilanol tiglate, understory

## Abstract

1. Flowering plants in tropical rainforests rely heavily on pollen vectors for successful reproduction. Research into pollination systems in tropical rainforests is dominated by canopy species, while subcanopy plant–pollinator interactions remain under‐represented. The microclimate beneath the rainforest canopy is characterized by low light levels and is markedly different from the canopy environment that receives more light energy.
2. We studied the floral attractants and floral visitors of a dioecious, subcanopy tree, *Fontainea picrosperma* (Euphorbiaceae), in the Wet Tropics bioregion of northern Queensland, Australia.
3. We found that wind pollination is rare and male and female flowers do not produce nectar. Female flowers are likely pollinated due to their perceptual similarity to pollen‐offering male flowers. Female flowers had the same scent profile as male flowers, and floral scent was an important floral attractant that acted to regulate pollinator behavior. The two most abundant scent compounds present in the floral bouquet were benzyl alcohol and 4‐oxoisophorone. These compounds are ubiquitous in nature and are known to attract a wide variety of insects. Both day‐time and night‐time pollinators contributed to successful pollen deposition on the stigma, and diurnal flower visitors were identified from several orders of insects including beetles, flies, predatory wasps, and thrips. *Fontainea picrosperma* is therefore likely to be pollinated by a diverse array of small insects.
4. Synthesis. Our data indicate that *F. picrosperma* has a generalist, entomophilous pollination syndrome. The rainforest subcanopy is a distinctive environment characterized by low light levels, low or turbulent wind speeds, and relatively high humidity. Female flowers of *F. picrosperma* exhibit cost‐saving strategies by not producing nectar and mimicking the smell of reward‐offering male flowers. Insects opportunistically forage on or inhabit flowers, and pollination occurs from a pool of small insects with low energy requirements that are found beneath the rainforest canopy.

## Graphical abstract

Pollinators beneath the rainforest canopy occupy a unique microclimate that is markedly different from the canopy environment that receives more light energy. We studied the floral attractants and floral visitors of a dioecious, subcanopy rainforest tree, *Fontainea picrosperma* (Euphorbiaceae), in northern Australia. *Fontainea picrosperma* is likely to be pollinated by small insects that opportunistically feed on or inhabit the flowers that offer poor rewards in the shaded subcanopy.

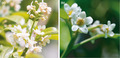

## 1 INTRODUCTION

Many angiosperms in tropical rainforests exhibit breeding mechanisms that promote outcrossing and rely almost exclusively on animals for pollination (Bawa, Perry, et al., 1985; Ollerton et al., 2011). Thus, pollination in rainforest ecosystems largely depends on animal abundance, distribution, and the flight behavior of flying insects (Aguilar et al., 2008; House, 1992, 1993; Stacy et al., 1996). Mutualistic plant–pollinator interactions help to govern and maintain the rich biodiversity in tropical rainforests which are renowned for their high levels of tree species diversity and animal pollinator diversity (Boulter et al., 2006). Generalized pollination syndromes are common in tropical rainforests, presumably because generalization is favored in species that promote outcrossing and have no reproductive assurance (Bawa, Perry, et al., 1985; Corlett, 2004; Vamosi et al., 2013).

Pollinator assemblages vary across tropical rainforest communities geographically and among vertical forest strata within rainforests. For example, trees beneath the canopy in Costa Rica receive more visits by pollinators such as hummingbirds and beetles than canopy trees. In contrast, canopy species receive more visits from medium‐ to large‐sized bees (Bawa, 1990). Bee assemblages are also known to differ between strata in the Brazilian Tropical Atlantic Rainforest (Ramalho, 2004). Vertical strata are defined as understory which includes plant species <5 m in height; subcanopy that is comprised of trees >5 m tall but less than canopy stature at reproductive maturity; and canopy species which includes trees flowering at the top of the forest, including emergent trees (Kress & Beach, 1994). Temporal availability of floral resources as well as differences between plant species composition can result in differences in pollinator or floral visitor assemblages among vertical strata. Moreover, the rainforest environment below the canopy is markedly different to the forest canopy where the high light energy input results in greater resource availability and allows tree species to produce highly productive flowers (Appanah, 1991; Roubik, 1993). The total radiation reaching the understory in closed tropical rainforests is 2–3 percent of that reaching the canopy (Mabberley, 1992). In addition, the microclimate beneath the canopy has high relative humidity, stable diurnal air temperatures, and low wind speeds or turbulent wind patterns compared to the canopy (Corlett, 2004; Kato, 1996; New, 2018). These conditions can pose challenges for pollinating insects and can reduce the effectiveness of aerial pollen transport (Corlett, 2004; Kato, 1996).

Most of the floristic diversity is found beneath the canopy of tropical rainforests (Ashley, 2010; Bawa, Bullock, et al., 1985; Hubbell, 2005). Studies of understory or subcanopy species in the Neotropics have been dominated by palm species where a variety of beetles, bees, flies, and orthopterans are attracted to the flowers (Aguirre et al., 2011; Borchsenius et al., 2016; Knudsen et al., 1999; Listabarth, 1993; Luna et al., 2005). Similarly, diverse taxa have been documented as flower visitors and pollinators in the understory or subcanopy in other rainforest environments where flowers are commonly visited by a range of small insects (Aguirre et al., 2011; Berecha et al., 2015; Borchsenius et al., 2016; Devy & Davidar, 2006; Kato, 1996; Momose et al., 1998). Tree species that occupy the rainforest beneath the canopy often possess small, white or green flowers, with small amounts of pollen and/or nectar (Boulter et al., 2006; Renner & Fell, 1993; Machado & Lopes, 2004). The poor floral rewards offered potentially reflect the low light energy environment of the rainforest subcanopy as plant resource availability is affected by the amount of light energy available (Appanah, 1991; Bawa, Bullock, et al., 1985). In turn, rainforest, subcanopy plant species may be unable to support the energy requirements of large pollinators due to the energetic costs associated with flower and nectar production (Appanah, 1991; Borrell, 2005; Pyke, 1991). Scent, rather than visual attraction, may be an important attraction signal in the shaded rainforest beneath the canopy (Williams & Adam, 2010). However, generalizations between floral scent compounds and pollination by different functional groups of insects can be difficult to render because of the lack of literature available (Cordeiro et al., 2019; Raguso, 2008).

*Fontainea picrosperma* C.T. White (Euphorbiaceae) is a dioecious, subcanopy tree endemic to the Australian Wet Tropics bioregion in upland tropical rainforests on the Atherton Tablelands, Queensland, Australia. *Fontainea picrosperma*'s current distribution has been disrupted by anthropogenic habitat fragmentation primarily due to agricultural expansion, but also due to urban settlements. The Australian Wet Tropics makes a significant contribution to global biodiversity, harboring 16 of the 28 near‐basal (or “primitive”) lineages of flowering plants in addition to a high level of species endemism (Metcalfe & Ford, 2009). Despite this significance, there are few published studies of flower visitors in this bioregion and even fewer empirical assessments of successful pollinators (Boulter et al., 2009). Moreover, relatively few studies globally have focused on the pollination systems of plant species in the subcanopy, an environment that is markedly different to the rainforest canopy, and our understanding of plant–animal interactions in subcanopy rainforest species is sparse.

The pollination biology of *F. picrosperma* is also of significant commercial interest because its fruit contains the small molecule natural product, tigilanol tiglate (Boyle et al., 2014), which has recently been approved as a veterinary oncology product as a therapeutic for canine mast cell tumors (De Ridder et al., 2020) and is in clinical development as a local treatment of solid tumors in humans (Panizza et al., 2019). Tigilanol tiglate is not synthetically tractable and consequently relies on harvest of plantation‐grown *F. picrosperma* fruit for its production. As apomixis (asexual reproduction) is not a significant means of reproduction in *F. picrosperma* (Grant et al., 2017), it is important to understand how pollen is delivered to the stigma. The aim of this study is to examine the floral attractants and flower visitors of a dioecious, subcanopy rainforest species, *F. picrosperma*. We asked the following questions: (a) What are the modes of pollen dispersal and deposition of *F. picrosperma*? (b) What are the main floral attractants in this subcanopy rainforest species? (c) What animals are attracted to the flowers in a subcanopy environment? Specifically, we ascertained whether wind is a potential pollination method, determined differences in the species' pollen deposition between nocturnal and diurnal pollinators, investigated floral attractants, and characterized the floral scent profile. We carried out flower observations, collected in‐flower visitors, and used scent lures to trap insects attracted to the main compounds present in *F. picrosperma*'s floral bouquet.

## 2 METHODS

### 2.1 Study species

**FIGURE 1:**
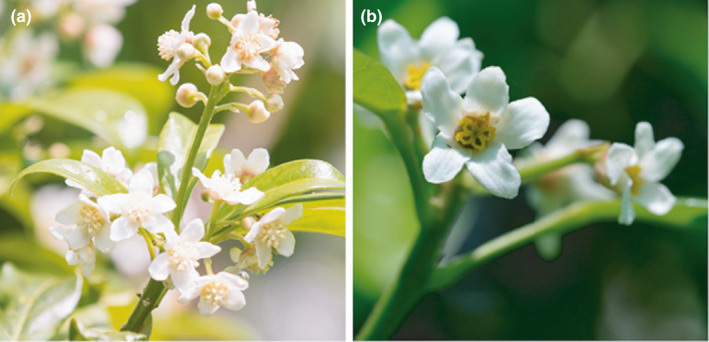
*Fontainea picrosperma* inflorescences. (a) Male flowers. (b) Female flowers

*Fontainea picrosperma* is a subcanopy tree that grows up to 25 m tall (Jessup & Guymer, 1985). It is endemic to the mesophyll and notophyll vine forests on the Atherton Tablelands, north Queensland, Australia. Male and female individuals possess inflorescences with small, white flowers that have an unspecialized structure and an open access receptacle (Figure 1; see also Grant et al., 2017). However, flowering phenology differs between sexes and individual female flowers open for significantly longer than individual male flowers (Grant et al., 2017). Flowering occurs simultaneously between individuals of subpopulations from September to November. The red drupaceous fruit (up to 3 cm diameter) ripen in December and January and are primarily dispersed by gravity. Natural stands of *F. picrosperma* therefore are not uniformly distributed; they form small, isolated but dense clumps (2–10 m intertree spacing) with ∼50:50 male:female ratios (Grant et al., 2017; Lamont et al., 2016).

*Fontainea picrosperma* has a geographically restricted natural range, and its distribution has been heavily influenced by both natural and anthropogenic habitat fragmentation (Lamont et al., 2016). The locations of the populations included in this study were Boonjie and Evelyn Highlands as described in Lamont et al. (2016). The subpopulations of “Evelyn Highlands 1” and “Evelyn Highlands 2” are from the same refugial population approximately 3.5 km apart. Boonjie and Evelyn Highlands are the two largest, continuous rainforest areas where *F. picrosperma* occurs. Data were collected over three flowering seasons in 2014, 2015, and 2016.

### 2.2 Pollen dispersal and deposition

To determine if wind contributes to pollination, microscope slides smeared with a thin layer of Vaseline™ the size of a cover slip (300 mm^2^) were hung from three male and three female flowering *F. picrosperma* trees (three slides per tree) in Evelyn Highlands 2. Slides were installed 1.5–3 m from the ground for 24 hr in the zone where flowers occur. New slides were installed each day for three replicate days. Once the slides were removed, the area of Vaseline was stained with Calberla's solution to detect pollen. The number of *F. picrosperma* pollen grains within the area of Vaseline™ was counted using a compound light microscope (Leica M125, Leica Microsystems, X 40).

Pollinator exclusion experiments were carried out in the natural population at Evelyn Highlands 2 to assess the pollen deposition rate of diurnal versus nocturnal pollinators. There were three treatments: (a) control, where inflorescences were open to both diurnal and nocturnal flower visitors; (b) diurnal, where inflorescences were open to insect visitors during the day (0600–1800 hr); and (c) nocturnal, where inflorescences were open to insect visitors during the night (1800–0600 hr). Inflorescences were enclosed at bud stage in a fine mesh (0.5 × 1.0 mm) bag fastened with a peg to exclude flower visitors, and the number of initial starting buds was counted. Five trees each with two to three replicate inflorescences for each treatment were bagged. Bags were removed or replaced each morning and evening for the duration of the experiment. Flowers were removed when the stigmas and petals began to shrivel and brown or were otherwise removed when subject to treatment for 10 days. Once removed, flowers were individually stored in fixative (25% glacial acetic acid, 75% ethanol; v,v). The number of pollen grains present on the stigma was then counted for open, diurnal, and nocturnal treatment groups using a fluorescence microscope (Leica DM5500B, Leica Microsystems) after staining with decolorized aniline blue (Kho & Bear, 1968; Shepherd, 2000).

### 2.3 Floral attractants

Five female trees and four male trees were sampled for diurnal nectar secretion from the natural population, Evelyn Highlands 2. Individual flowers were tested for nectar rewards using a wash method following Morrant et al. (2008). This method is recommended for species with low floral nectar volumes and can measure nectar that accumulates from trichomal nectaries. Unopened buds were tagged and then bagged to exclude flower visitors for the duration of the experiment. One flower per tree was measured at various times on each day of sampling, when the flower was 0, 2, 3, and 5 days old. Each flower was placed into a vial containing 0.5 ml of distilled water. The sample was manually agitated for 5–10 min. The sugar concentration was measured using a hand‐held BRIX refractometer (ATAGO PAL‐S). This is a destructive method; therefore, a flower sampled at 5 days after opening, for example, remained bagged for the 5 days.

We identified volatile compounds emitted from *F. picrosperma* flowers from potted plants that were sourced from natural populations and housed in the glasshouses at the University of the Sunshine Coast (USC), Sippy Downs, Australia. In the glasshouse, environmental conditions including light, temperature, and moisture were constant for each plant. We assessed the diurnal floral volatile emission profile over time and consistency of volatiles between the sexes. Whole flowers were removed from *F. picrosperma* plants 1–2 mm below the base of the flower, for ease of headspace volatile collection. This method does increase the risk of obtaining higher levels of green leaf volatiles that are characteristic of wounded foliage (e.g., Arimura et al., 2001; Grison et al., 1999). However, the short sampling time of SPME minimizes the collection of artifacts due to damage to the plant (Flamini et al., 2003). Six male and six female trees were used in the experiment. Flowers were sampled at different ages relative to opening (male 0, 1, 2, and 3 days; female 0, 1, 2, 3, 5, 7, 9, and 11 days). The differences in sampling ages between male and female flowers reflect the different male and female flower opening times. Three to six replicate flowers were sampled per flower age per sex (*n* = 23 males flowers and *n* = 24 female flowers in total). Flowers were removed between 0900 and 1500 hr and placed in a 10‐ml septum cup vial to allow volatile odors to equilibrate for 1 hr at room temperature (22℃). A background control of the glasshouse and sample vials was also sampled each day of floral volatile testing. Volatiles from each sample were then collected using a solid‐phase microextraction (SPME) holder (Augusto & Luiz Pires Valente, 2002). For each sample, the Supelco fiber coated with polydimethylsiloxane (PDMS, 100 µm) was manually inserted into the headspace chamber and exposed for 25 min at 22℃.

Volatile analysis was performed on a PerkinElmer Clarus 580 GC‐MS. The column used was an Elite‐5MS (30 m × 0.25 mm × 0.25 μm). The helium carrier gas had a constant flow of 1 ml/min. The SPME fiber was manually inserted into the injection port fitted with a splitless liner at 200℃ and maintained for 2 min to allow the adsorbed volatiles to thermally desorb onto the GC column. The split ratio was shut from −0.5 to 2 min and then open at 30:1. The temperature program was operated at 40℃ for 2 min, ramping at 10℃/min until 210℃ and holding for 1.5 min. The mass spectrometer analyzed a mass range from 40 to 250 (m/z), from 1 to 20.5 min at 70 eV. Compounds were identified by comparison of (a) retention times to authentic standards, (b) Arithmetic Index (AI) (Adams, 2007), and (c) mass spectra of National Institute of Standards and Technology (NIST 08) Mass Spectral library.

Compounds present at similar abundance in the control samples were considered to be contaminants and were excluded from the analysis. Proportional abundance (relative amounts with respect to aggregate peak areas, excluding contaminants) of the individual constituents was calculated based on peak area measurements and expressed as a percent of the sum total peak area.

### 2.4 Flower visitors

Two *F. picrosperma* populations, Evelyn Highlands 2 and Boonjie, were surveyed for diurnal flower visitors. Three male trees and three female trees for each population were each observed for a 10‐min period every 2 hr (0820–1720 hr). Observations were conducted on sunny days with light winds during times conducive to diurnal insect activity. A total of 25 observation hours (5 days) were carried out in Evelyn Highlands 2 and 20 observation hours (4 days) were carried out in Boonjie. Details of insect visitor behavior were also recorded during the observation time. Individual visitors were collected where possible and stored in 70 percent ethanol. Means and standard errors of the number of visits per observation period were calculated for each order of insect. Sticky traps were installed in male and female trees adjacent to inflorescences during the night to detect for nocturnal insect activity; however, no insects were captured.

“In‐flora” visitors were collected by enclosing individual inflorescences in sealable plastic bags and clipping the stem at the point of closure of the bag. Inflorescences were opportunistically sampled, and the specimens collected in the samples were later identified. The number of open flowers and the number of buds were recorded for each inflorescence. The mean numbers of Thysanoptera (thrips) per inflorescence were calculated for each sex.

We trapped insects using a scent lure to ascertain what types of insects were attracted to the floral scent compounds emitted by *F. picrosperma* flowers. Field trap experiments were carried out during the flowering season in Evelyn Highlands 1. Two types of insect traps were used to target a range of insects. Black plastic colored collision traps (Sankei Chemicals Co. Ltd.; 25 cm diameter, 42 cm height) and commercial white sticky traps (9 × 15 cm) were installed together as pairs in trees approximately 2 m above ground. Collision traps were comprised of plastic vertical panels into which flying insects collide and fall into a trough filled with water. Panels were treated with a fluoropolymer, Fluon, to enhance their efficiency (Graham et al., 2010). Sorbic acid (1 g/L) was added to the collision trap to prevent decay of the insects, and small quantities of a surface‐active agent (neutral detergent) were also added to the water in the trap. Scent baits were attached with string to the collision traps so that they hung just above the water surface. The scent baits were attached to the top of one side of the sticky trap. Control traps did not have a scent lure. Six pairs of traps baited with a scent lure and six pairs of traps without a scent lure (control) were installed in separate nonflowering trees for 1 week. The distance between each pair of traps was a minimum of 15 m and a minimum of 10 m from the nearest flowering *F. picrosperma*.

A synthetic mix of the main floral scent compounds detected in the SPME scent analysis was used as a scent lure. The compounds included in the scent lure were *cis* 3‐Hexen‐1‐ol acetate; Benzyl alcohol; Ocimene isomer mixture (3,7‐Dimethyl‐1,3,6‐octatriene); Methyl benzoate; 4‐oxoisophorone (2,6,6‐Trimethyl‐2‐cyclohexene‐1,4‐dione); and Benzyl acetate all obtained from Sigma‐Aldrich, Inc. The compounds were added to the scent lure according to the average proportional abundance (%) that was detected in the GC‐MS analysis based on a female flower at day three of opening. Different dilutions of the scent lure compound mixture were tested on the GC‐MS to confirm the scent composition relative to the female flower scent profile and select a solution that could be maintained over the one‐week installation period. A scent lure was devised for dispensing scent chemicals using a mini snap lock bag containing two filters that were impregnated with 500 µl using 1:10 dilution of scent solution with paraffin oil. Captured insects were preserved in a vial containing 70 percent ethanol for later identification.

### 2.5 Statistical analysis

We tested for differences in the sum total of all detected compounds emitted between different flower ages within sex using a generalized linear model (GLM) with a negative binomial distribution and a log link function (package “MASS”; Venables & Ripley, 2002). Where differences were detected, we applied a Tukey's HSD test. For analyses of semiquantitative (i.e., relative amounts of scent compounds within a flower) differences in scent between sex, total peak area of compounds were fourth‐root transformed before analysis. We calculated the Bray–Curtis similarity index and performed a permutational multivariate analysis of variance fitted to the distance matrices (PERMANOVA) (999 permutations) using the Adonis function (package “vegan”; Oksanen et al., 2016). We first analyzed the combined ages 0–3 days and then compared each age separately to test for differences between sex as the flower aged (package “pairwise.adonis”; Martinez Arbizu, 2019). Analyses were performed in R v.3.5.1 (R Development Core Team).

We tested for treatment (diurnal; nocturnal; open to flower visitors) effects on the number of pollen grains detected on a stigma using a GLM with a negative binomial distribution and a log link function to correct for overdispersion due to inflated zeros in the data (package “MASS”; Venables & Ripley, 2002). Differences between treatments were assessed using Tukey's HSD post hoc test (package “multcomp”; Hothorn et al., 2008). Analyses were performed in R (v.3.5.1).

Counts of individual morphospecies found in paired collision and sticky traps were combined. We conducted a Mann–Whitney *U* test (SPSS v 24) to determine if there were significant differences (*p* < 0.05) between the number of individuals of each morphospecies in the control and scent lure traps. Only morphospecies with more than 10 individuals in the insect traps were included in the analysis (*n* = 29). Morphospecies with significant differences between treatment and control (*n* = 5) were identified to genus level. We also combined the number of individuals of each morphospecies within an order and tested for significant differences between treatments (*p* < 0.05, Mann–Whitney *U* test). Analyses were performed using IBM SPSS v 24.

## 3 RESULTS

### 3.1 Pollination

To track the movement of pollen, we tested for the potential of wind pollination and the effectiveness of day and night pollinators. Few airborne pollen grains were captured in the natural population (Table 1).

**TABLE 1:** Mean number of airborne pollen grains detected per tree over a 24‐hr period on the microscope slides in a natural *Fontainea picrosperma* population, Evelyn Highlands 2 (standard error in parentheses), and the approximate number of equivalent pollen grains per stigma area

Sex	Number of airborne pollen grains	Equivalent number of pollen grains per stigma area
Male	1.18 (0.30)	0.022
Female	1.85 (0.40)	0.034

**FIGURE 2:**
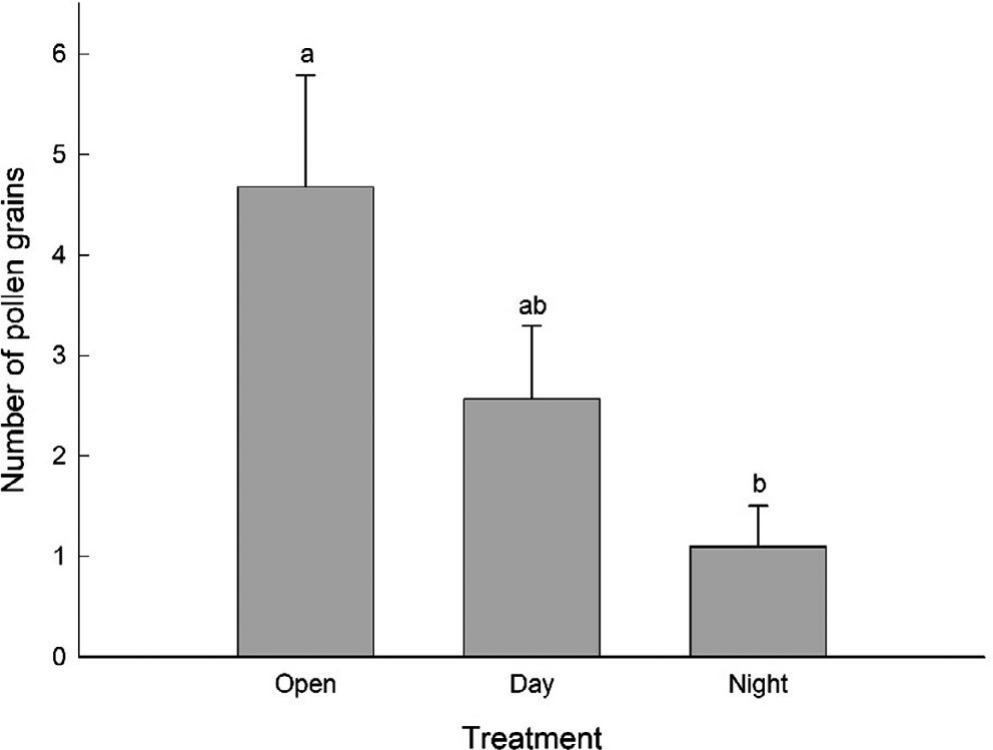
Mean number of pollen grains counted on the stigma of female *Fontainea picrosperma* flowers. Open, inflorescences not bagged (*n* = 28); Day, inflorescences open to pollinators during the day (0600 hr–1800 hr; *n* = 23); Night, inflorescences open to pollinators during the night (1800 hr–0600 hr; *n* = 21). Treatments with different letters are significantly different (GLM, *p* < 0.05)

Pollination treatments affected pollen deposition in the natural population, Evelyn Highlands 2 (Figure 2). Significantly more pollen grains were found on open (control) flowers than on flowers that were only exposed to nocturnal visitors (GLM, *p* = 0.005; Figure 2). There was no significant difference in the number of pollen grains between open flowers and flowers that were exposed to visitors during the day (GLM, *p* = 0.373; Figure 2) or between flowers that were only open to visitors during the day and flowers only open to visitors during the night (GLM, *p* = 0.183; Figure 2).

### 3.2 Floral attraction

There was no diurnal nectar secretion in *F. picrosperma* flowers. Nor was there any visible accumulation of liquid on the petals, stamens or pistils, or in the base of the receptacle of any of the flowers examined at dawn or dusk. Since we did not test for nocturnal nectar production specifically, it is unclear if the flowers secreted and reabsorbed nectar during the night.

Benzyl alcohol and 4‐oxoisophorone (2,6,6‐Trimethyl‐2‐cyclohexene‐1,4‐dione) were the two major scent compounds present in both male and female flowers (Table 2). The next most relatively abundant compounds were β‐Ocimene (unknown isomer), Methyl benzoate (Benzoic acid, methyl ester), and *cis*‐3‐Hexen‐1‐ol acetate. Trace amounts (generally ≤1%) of compounds that were detected in *F. picrosperma*'s floral scent bouquet included 1,1‐Dimethyl‐3‐methylene‐2‐vinylcyclohexane, 1,4‐Cyclohexanedione, β‐Cyclocitral, Benzyl acetate (Acetic acid, phenylmethyl ester), Benzaldehyde, Methyl salicylate (Methyl 2‐hydroxybenzoate), Hexyl acetate (Acetic acid, hexyl ester), and Geranyl isovalerate ((E)‐3,7‐Dimethyl‐2,6‐octadienyl 3‐methylbutanoate) (Table 2).

**TABLE 2:** Floral volatile compounds detected by GC‐MS of male and female flowers aged from 1 to 3 days of *Fontainea picrosperma* during the day

Compound class	Compound name	Relative %	
		Male	Female
Terpenes			
Monoterpenes			
	β‐Ocimene, unknown isomer^a^	3.06 (0.5)	4.07 (0.5)
Irregular terpenes (Apocarotenoids and related compounds)			
	4‐oxoisophorone^a^	43.24 (3.4)	31.87 (2.4)
	2,2,6‐Trimethyl‐1,4‐cyclohexanedione	0.64 (0.1)	1.03 (0.4)
	β‐Cyclocitral^b^	0.18 (0.0)	0.21 (0.0)
	Geranyl isovalerate	0.09 (0.0)	0.10 (0.0)
	1,1‐Dimethyl‐3‐methylene‐2‐vinylcyclohexane	0.01 (0.0)	0.11 (0.0)
Benzenoids			
	Benzyl acetate^a^	0.90 (0.1)	1.32 (0.1)
	Benzyl alcohol^a^	31.28 (2.3)	49.26 (2.0)
	Benzaldehyde^b^	1.06 (0.1)	1.06 (0.1)
	Methyl benzoate^a^	17.91 (2.8)	6.94 (1.3)
	Methyl salicylate^b^	0.23 (0.0)	1.32 (0.6)
Aliphatics (Volatile fatty acid derivatives)			
	*cis*‐3‐Hexen‐1‐ol acetate^a^	1.12 (0.2)	2.40 (0.3)
	Hexyl acetate^b^	0.27 (0.0)	0.31 (0.0)

Note

Scent compounds are listed according to compound class. Mean relative percent of each compound is presented using data from flowers aged from 0 to 3 days. Standard error in parentheses.

^a^ Compounds were identified based on GC retention times and mass spectra of standard compounds purchased from Sigma‐Aldrich.

^b^ Compounds were identified using the mass spectrum and AI (arithmetic retention index) (Adams, 2007). The remaining compounds were tentatively identified according to their mass spectral and retention index data in the National Institute of Standards and Technology (NIST 08) Mass Spectral library.

**FIGURE 3:**
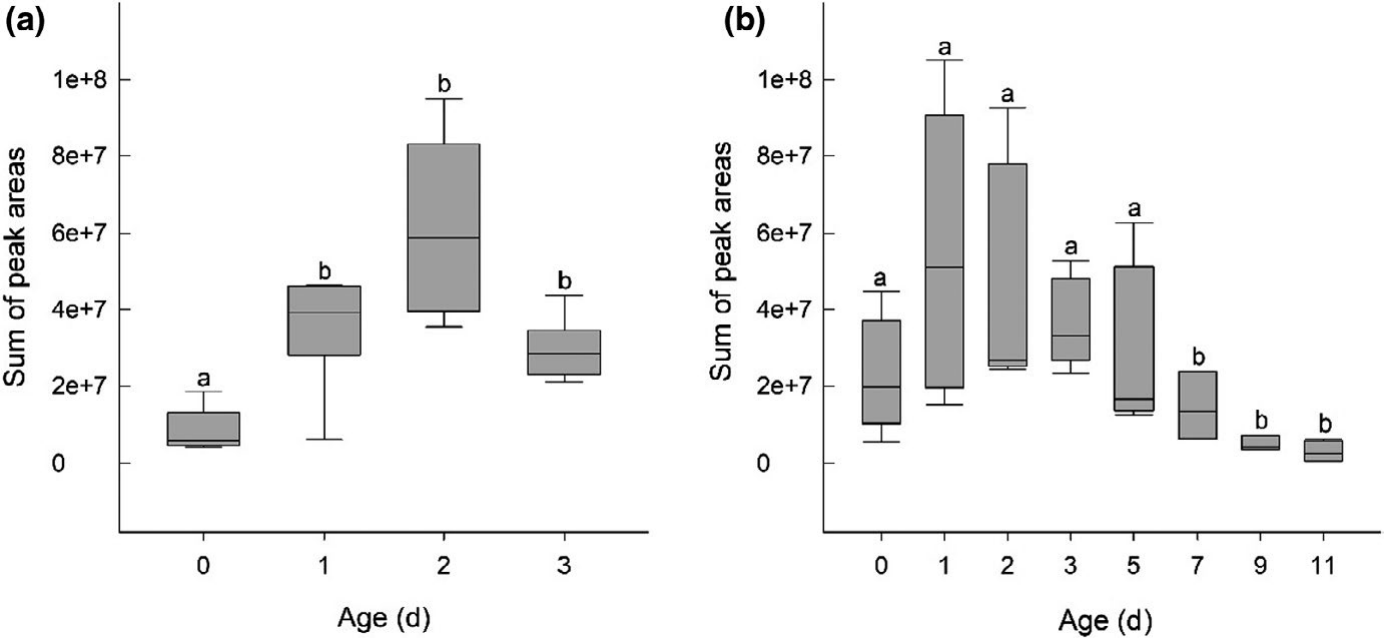
Emission patterns of the sum total of aggregate peak areas of all compounds across the age of flowers of *Fontainea picrosperma* in (a) male flowers and (b) female flowers

Male flowers emitted significantly less scent at 0 day than male flowers at age 1, 2, or 3 days based on sum of total peak area (GLM, *p* < 0.05; Figure 3). The senescing stage of female flowers was characterized by a drop in overall volatile emissions (Figure 3). Older female flowers (>5 days) emitted significantly less scent than flowers aged 0–5 days (GLM, *p* < 0.05; Figure 3).

**FIGURE 4:**
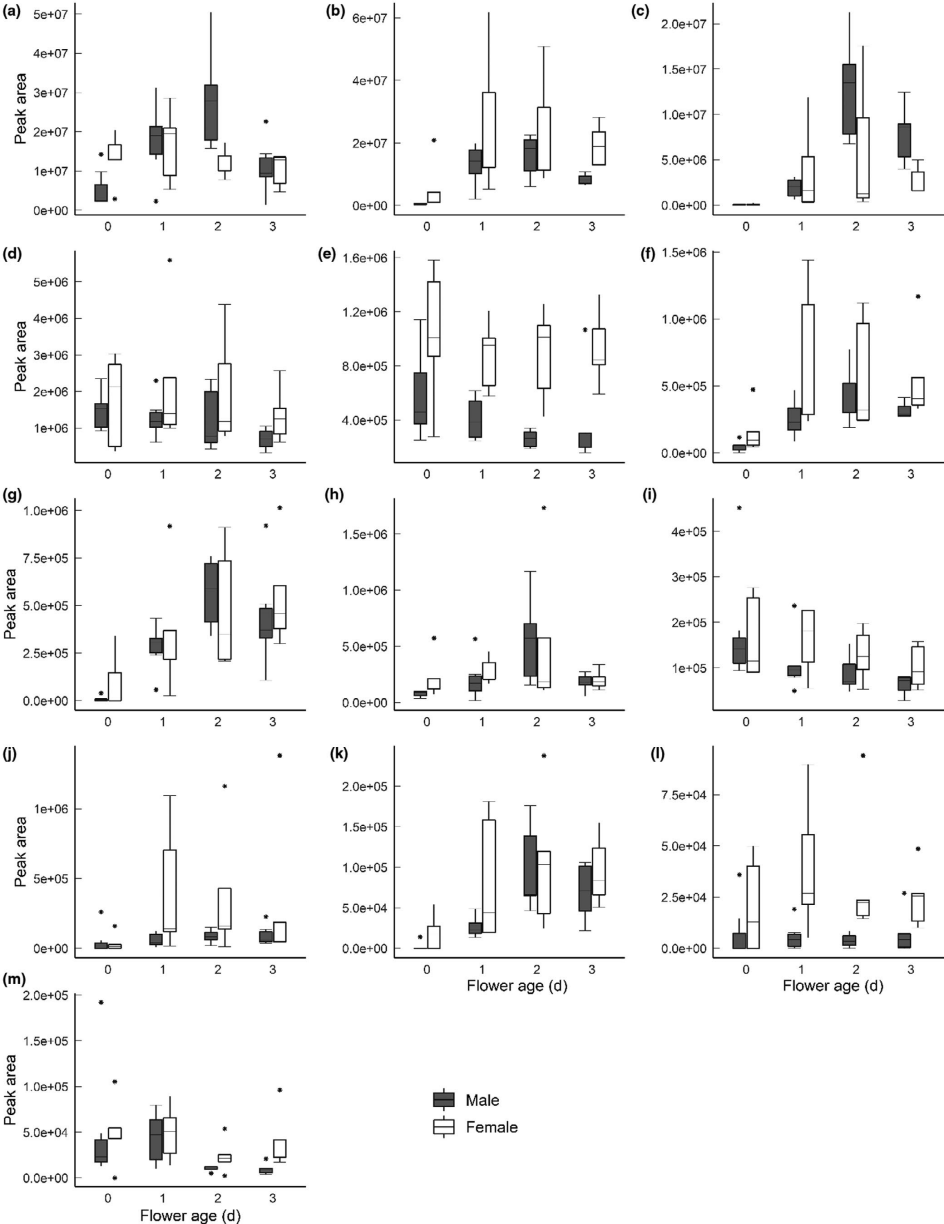
Peak area of the detected floral volatile compounds from male and female *Fontainea picrosperma* flowers for each age 0–3 days. (a) 4‐oxoisophorone, (b) Benzyl alcohol, (c) Methyl benzoate, (d) β‐Ocimene (unknown isomer), (e) *cis*‐3‐Hexen‐1‐ol acetate, (f) Benzyl acetate, (g) Benzyl aldehyde, (h) 2,2,6‐Trimethyl‐1,4‐cyclohexanedione, (i) Hexyl acetate, (j) Methyl salicylate, (k) β‐Cyclocitral, (l) 1,1‐Dimethyl‐3‐methylene‐2‐vinylcyclohexane, and (m) Geranyl isovalerate. For each compound, the graphs show the median (quartiles, minimum–maximum) of the total peak area (*n* = 5–6 for male flowers and *n* = 3–5 for female flowers)

Pairwise comparisons revealed no significant differences in the bouquet of floral volatile emissions (peak area) between male and female flowers when combined across ages of 0–3 days (PERMANOVA, *R* ^2^ = 0.053, *p* = 0.082). When the different daily ages of flowers were analyzed individually, pairwise comparisons revealed significant differences between male and female flowers at age 0 days (PERMANOVA, *R* ^2^ = 0.218, *p* = 0.045) and at age 3 days (PERMANOVA, *R* ^2^ = 0.372, *p* = 0.002). There were no significant differences between male and female flowers at age 1 day (PERMANOVA, *R* ^2^ = 0.172, *p* = 0.181) and 2 days (PERMANOVA, *R* ^2^ = 0.265, *p* = 0.071). The aggregated peak area of individual compounds differed over the age of the flower (Figure 4).

### 3.3 Flower visitors

**FIGURE 5:**
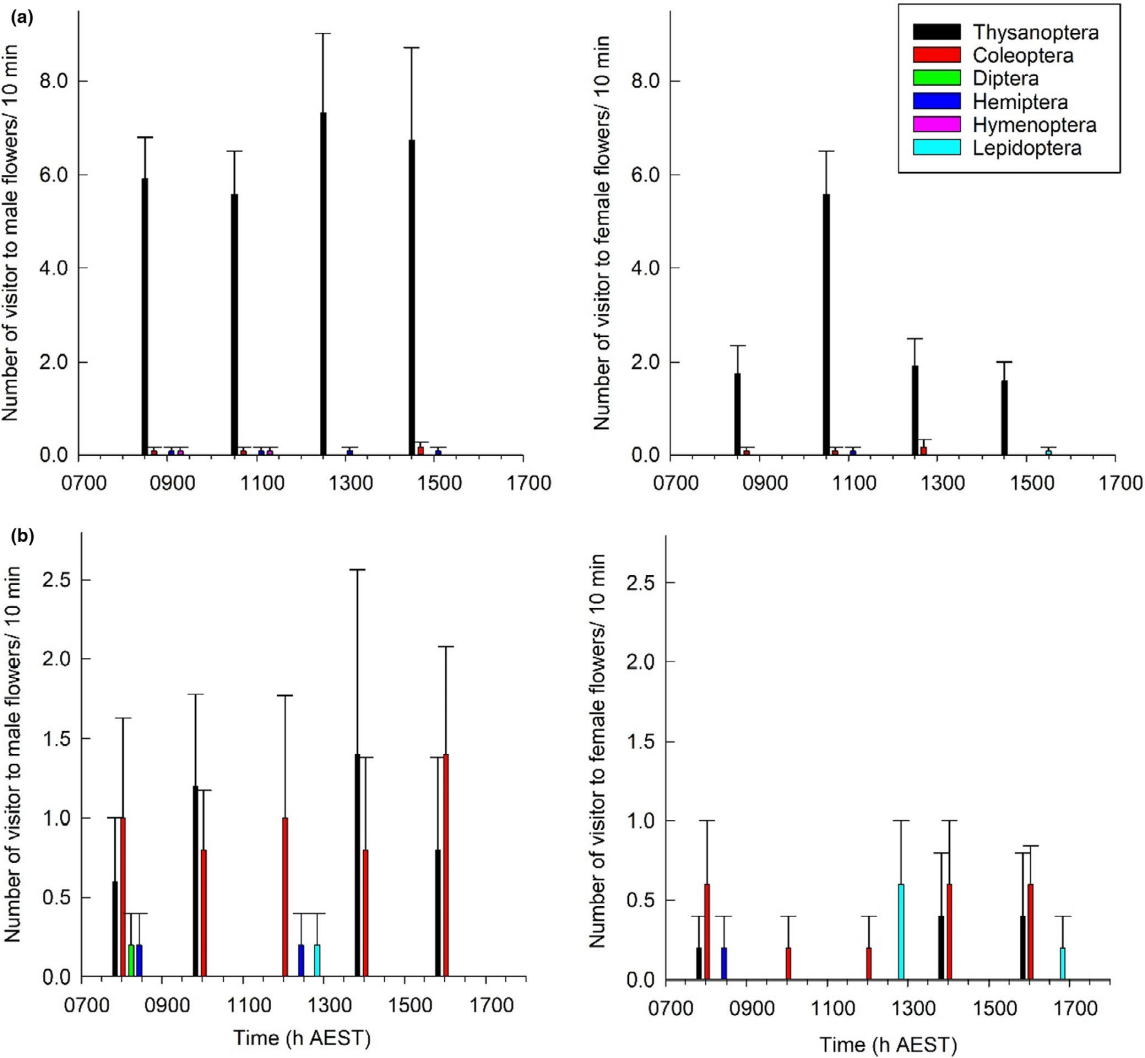
Diurnal variation in number of flower visitors to *Fontainea picrosperma* in (a) Boonjie and (b) Evelyn Highlands 2. Mean and standard error for number of visits for each observation period in male trees and female trees

Flowers were most frequently visited by insects from the orders Thysanoptera (thrips) and small (<1 cm in length) Coleoptera (beetles) (Figure 5). More thrips were observed in the Boonjie subpopulation compared to the Evelyn Highlands 2 subpopulation. Thrips were not observed departing from the inflorescence during the day and therefore behaved as permanent inhabitants according to the criteria in Listabarth (1993). Beetles were often observed crawling on all parts of the inflorescence or chewing on flower parts including stigmas, anthers, and petals. The Lepidoptera observed in Evelyn Highlands 2 were all caterpillars, except one unidentified butterfly that briefly landed on the petals of a female flower and flew away without interacting with the stigma.

The most abundant insects captured within sampled inflorescences were thrips (Table 3). A range of small (<1 cm in length) “in‐flora” flower visitors from whole inflorescences were also present (Table 4). Aside from mosquitoes (*n* = 2), only a single specimen of each insect (*n* = 1) was collected in the flowers.

**TABLE 3:** Mean number of Thysanoptera captures from in‐flora collections of *Fontainea picrosperma* inflorescences in the natural populations at Boonjie, Evelyn Highlands 1, and Evelyn Highlands 2 per inflorescence

Population	Number of inflorescences		Number of Thysanoptera	
	Male	Female	Male	Female
Boonjie	12	12	4.92 (1.3)	7.17 (2.2)
Evelyn Highlands 1	9	3	2.89 (0.8)	2.00 (1.2)
Evelyn Highlands 2	5	1	0.20 (0.2)	0.00 (0.0)

Note

Standard error shown in parentheses.

**TABLE 4:** Flower visitors of *Fontainea picrosperma* based on insect captures during observations and in‐flora collections of insects and mites in the natural populations at Boonjie, Evelyn Highlands 1, and Evelyn Highlands 2

Location	Sex	Number of flowers	Order	Identification^a^	Common name
Flower visitors captured within inflorescences					
Boonjie	F	6	Hemiptera	Fulgoroidea	Leaf hopper nymph
Boonjie	F	3	Hemiptera	Coccoidea	Scale insect
Boonjie	M	8	Coleoptera	Nitidulidae	Sap beetle
			Coleoptera	Curculionidae	Weevil
Boonjie	M	8	Hymenoptera	Encyrtidae	Parasitic wasp
Boonjie	M	9	Sarcoptiformes	Oribatida	Mite
Evelyn Highlands 1	M	3	Hymenoptera	Scelionidae	Parasitic wasp
Evelyn Highlands 1	M	4	Diptera	Culicidae	Mosquito
Evelyn Highlands 1	M	5	Hymenoptera	Platygastridae	Parasitic wasp
			Collembola	—	Springtail
Flower visitors captured during observations					
Evelyn Highlands 2	M	—	Coleoptera	Mordellidae	Pin tail beetle
Evelyn Highlands 2	F	—	Coleoptera	Curculionidae	Weevil
Evelyn Highlands 2	M	—	Coleoptera	Chrysomelidae: Galerucinae: Alticini	Flea beetle
Evelyn Highlands 2	F	—	Lepidoptera	—	Flower caterpillars

Note

The number of flowers present on each inflorescence is stated.

^a^ Levels of identification range from suborder (Oribatida) and superfamily to tribe.

**FIGURE 6:**
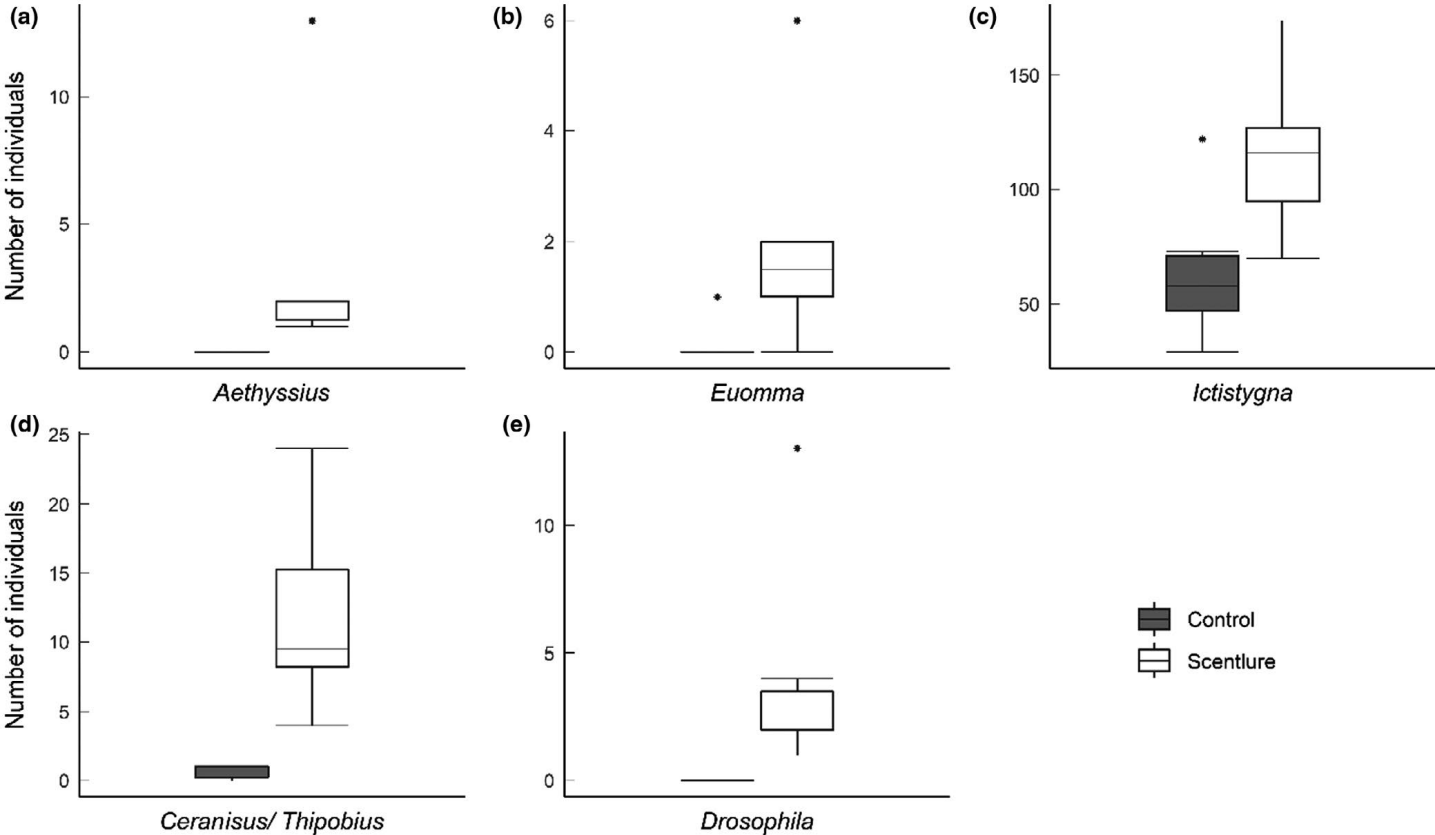
Insects (identified to genus level) found in significantly larger numbers in the scent lure traps compared to the control traps with no scent lure (*p* < 0.05, Mann–Whitney *U* test)

Five genera of insects were found in significantly greater numbers in the scent lure traps compared to the control traps in the Evelyn Highlands 1 population (*p* < 0.05, Mann–Whitney *U* test; Figure 6). Three were beetles including *Aethyssius* (Tenebrionidae: Alleculinae); *Ictistygna* (Anthicidae: Eurygeniinae); and another alleculine tentatively identified as *Euomma*. The other two genera were a minute (body length <1 mm) thrips parasitoid wasp that was either a species of *Ceranisus* or *Thripobius* (Eulophidae: Entedoninae) and *Drosophila* (Drosophilidae).

There were no significant differences between scent lure and control traps in any other taxa. When the number of individuals in each order was combined, there was a significant difference in the number of Lepidoptera between treatments (*p* = 0.034), with more present in the scent lure traps. There was no significant difference in any other order tested.

## 4 DISCUSSION

Our results indicate that floral scent is an important attractant for recruiting pollinators in *F. picrosperma*. The flowers of different sexes smell the same when pollen is available in male flowers and stigmas are receptive in female flowers. Neither male or female flowers appear to produce nectar and thus female flowers are likely to attract pollinators be mimicking the shape, color and scent of reward‐offering male flowers. The two main scent constituents in *F. picrosperma*, benzyl alcohol and 4‐oxoisophorone, contributed 78 percent of the relative floral emissions and are ubiquitous in floral scent profiles (Knudsen et al., 2006). A wide variety of small insect taxa were observed visiting flowers of both sexes, and both day and night visitors contributed to pollen deposition on the stigmas. Together with the unspecialized structure of *F. picrosperma* flowers (Grant et al., 2017), our data indicate that *F. picrosperma* has a generalist, entomophilous pollination syndrome.

### 4.1 Floral attraction

No diurnal nectar secretion was found in *F. picrosperma* flowers. Our results suggest that pollinators are likely deceived by female flowers that offer no obvious reward due to their perceptual similarity to pollen‐offering male flowers (Grant et al., 2017). Bakerian mimicry (where female flowers mimic male flowers and cheat pollinators out of a reward) can occur when females economize resources by not producing nectar (Renner, 2006; Thakar et al., 2003). Since nectar is costly in terms of seed production and photoassimilate production (Pyke, 1991; Southwick, 1984), the poor floral rewards offered potentially reflect the low light energy environment of the rainforest subcanopy (Appanah, 1991; Bawa, Bullock, et al., 1985).

Floral volatiles provide a way to attract pollinators, particularly in the shaded rainforest subcanopy where visual cues may be less important (Appanah, 1991; Knudsen et al., 1999; Koski, 2020; Williams & Adam, 2010). This occurs in other tropical species such as the understory palm *Geonoma macrostachys* (Borchsenius et al., 2016). The bouquet of floral volatile emissions did not differ between male and female flowers when combined across the ages of 0–3 days (*p* = 0.082). Thus, pollination likely occurs when insects are attracted by the scent of female flowers as they search for pollen‐offering male *F. picrosperma* trees.

The floral bouquet of male and female *F. picrosperma* flowers changes over time. We found that the volatiles differed between male and female flowers on the first day of opening and on day 3 of opening. Female flowers last significantly longer than do male flowers, and by age 3 days, female flowers are at the peak of receptivity. Alternatively, male anthers dehisce approximately one day after opening and begin to senesce at age 2–3 days (Grant et al., 2017). In addition, the total floral scent emitted was highest in male flowers when pollen was available with the anthers fully open and the highest in female flowers when the stigmas were receptive. Female flowers do not set fruit beyond 9 days (Grant et al., 2017), and the senescing stage of female flowers was characterized by an overall drop in the relative total of volatile emissions. Thus, *F. picrosperma* decreases scent production when pollen is not available or the stigma is not receptive and scent potentially regulates pollinator behavior across time to maximize reproductive success (Delle‐Vedove et al., 2017).

The two most abundant compounds in the floral scent profile of *F. picrosperma* comprised approximately 78 percent of the relative emissions from male and female *F. picrosperma* flowers (based on flowers aged from 1 to 3 days). These were the irregular terpene, 4‐oxoisophorone and the benzenoid, benzyl alcohol. 4‐oxoisophorone is the main floral volatile compound of species of *Buddleja*, a genus well known to attract butterflies and other insects (Chen et al., 2012, 2014, 2015). The compound also evokes antennal responses in moths, bees, and flies (Andersson, 2003; Guédot et al., 2008). Benzyl alcohol and methyl salicylate, also a constituent of *F. picrosperma*'s floral emissions, are among the most common compounds in floral scent, occurring in 56 percent and 57 percent of families studied, respectively (Knudsen et al., 2006). Benzyl alcohol is known to attract *Apis mellifera* (honey bees; Dötterl & Vereecken, 2010 and references therein). Benzyl alcohol, as well as several of the other main floral compounds of *F. picrosperma* such as benzaldehyde, benzyl acetate, and methyl salicylate are known to dominate the floral scent profile of plants with nocturnal anthesis and are associated with moth, hawkmoth, nocturnal bee, nocturnal beetle, and other generalist insect pollination (Etl et al., 2016; Raguso et al., 1996). Other benzenoids present in the floral bouquet of *F. picrosperma*, such as benzaldehyde, also have a widespread distribution and are thought to be functionally attractive to pollinators (Schiestl, 2010). The monoterpene β‐ocimene and the benzenoid methyl benzoate comprised a further 16 percent of the floral scent profile of *F. picrosperma*. β‐ocimene is the most commonly encountered scent compound in generalist plant–pollinator interactions (Dobson, 2006), and *cis*‐β‐ocimene has been shown to stimulate foraging behavior in bees (Eltz et al., 2006; Gong et al., 2015). Methyl benzoate is also known to attract bees (Dudareva et al., 2000; Schiestl & Roubik, 2003) and stimulate electroantennogram responses in moth antennae (Raguso & Light, 1998).

These results are one of the few studies on a species of Euphorbiaceae, which remains a poorly sampled family with regard to floral chemistry (Knudsen et al., 2006). Nevertheless, this study shows that the floral bouquet of *F. picrosperma* contains compounds that have a widespread distribution and are known to attract a wide variety of both diurnal and nocturnal insect pollinators.

### 4.2 Flower visitors

We determined that *F. picrosperma* flowers are visited by a diverse set of small insects. This is concordant with many other woody plants in Australian tropical and subtropical rainforest communities (Boulter et al., 2005; House, 1989; Webber et al., 2008; Williams & Adam, 1994; Worboys & Jackes, 2005) and with many species in rainforest communities elsewhere, for example, lowland dipterocarp forests (Kato, 1996; Momose et al., 1998). The floral traits of *F. picrosperma* suggest little adaptation to exploit specific pollinators (Grant et al., 2017). Both day and night visitors successfully deposited pollen on the stigma of female flowers. Small insect pollinators have low energy requirements and often move shorter distances compared to specialized insects, larger insects, or vertebrates that occur in the canopy (Appanah, 1991; Dick et al., 2008). This is supported by pollen gene flow data for *F. picrosperma* where pollen is found to disperse short distances (Grant et al., 2019).

Thrips were the most abundant insects observed and collected from both male and female *F. picrosperma* flowers. In other plant species, thrips inhibit the flowers and feed on the internal wall of the receptacle and pollen and contribute very little to cross pollination (Kondo et al., 2016). Their small body size means their movements can bypass the central stigma of female flowers (Irvine & Armstrong, 1990) and, combined with a weak flying ability, this could preclude thrips from efficient pollen transport because they are only able to carry small quantities of pollen. *Fontainea picrosperma* pollen grains are 40 µm in diameter (Grant et al., 2017), and pollen grain size in plants pollinated by thrips is usually <34 µm (Sakai, 2001). Thrips were the most common flower visitor observed in this study and by sheer numbers could contribute to pollination and successful reproduction in *F. picrosperma*. In addition, thrips may indirectly contribute to pollination by attracting predators or parasites. In this study, a specialist thrips parasitoid wasp, either a species of *Ceranisus* or *Thripobius* (Eulophidae), was significantly more attracted to the scent lure traps than the control traps with no scent. Other small parasitoid wasps including endoparasitoids of Coleoptera, Hemiptera, and Diptera (Austin et al., 2005) were found in flowers, and these orders of insects were observed visiting the flowers of both male and female trees.

Beetles (Coleoptera) were the second most commonly observed and collected visitors to *F. picrosperma* flowers. Beetles are thought to be critical in pollination in Australian tropical rainforests throughout the vertical strata and could pollinate up to one quarter of Australian tropical rainforest species (Irvine & Armstrong, 1990; Kitching et al., 2007; Wardhaugh et al., 2015; Webber et al., 2008; Worboys & Jackes, 2005). Thus far, no reliable pattern in floral scent chemistry unifies plants pollinated by tropical beetles (Dobson, 2006). Field observations suggest that the floral tissue provided some food reward targeted by chewing insects, which could cause incidental pollination. Beetles, for example, are known to visit flowers to feed on pollen, various floral tissues, and other floral exudates (Endress, 1996; Momose et al., 1998). *Ictistygna* sp. (Anthicidae: Eurygeniinae) was the most abundant species captured in the scent lure traps. Adult anthicid beetles are omnivorous, being known to consume small arthropods and pollen and some species within the family are known predators of stink bugs (Hemiptera: Pentatomidae) (Athey et al., 2019) and *Diaphania* spp. (Lepidoptera: Crambidae) (Júnior et al., 2012), though little is known about eurygeniine biology (Lawrence & Ślipiński, 2013). The two other beetle species belonged to the Tenebrionidae, a family that characteristically feed on dead vegetable or animal matter and living plant tissue, although a few normally predacious species are known (Watt, 1974).

Tropical rainforests are rich in dipteran species, and flies are capable of transporting significant quantities of pollen, usually short distances (House, 1989; Weiss, 2001). Significantly more drosophilid flies were captured in the scent baited traps than in the control traps. In other plant species studied where drosophilid flies were among the most abundant insect visitors, they carried little or no pollen and were inefficient or ineffective pollinators (Borchsenius et al., 2016). In this study, while drosophilid flies were attracted to the floral scent compounds emitted by *F. picrosperma* flowers, it is unclear if they are effective pollinators.

Pollination leading to successful fertilization is not always guaranteed by flower visitors, and further research is required to determine the pollination efficacy of different flower visitors to *F. picrosperma*. Nocturnal floral attraction and visitation could also warrant further research as our data primarily focused on diurnal activities. Nevertheless, we conclude that *F. picrosperma* is likely pollinated by an array of small insects such as thrips, beetles, flies, and predatory wasps.

This study provides further evidence for the assertion that subcanopy or understory tropical rainforest species with flowers that lack morphological specialization are commonly pollinated by a diverse array of small insects (Appanah, 1991; Bawa, Bullock, et al., 1985; Devy & Davidar, 2006; Momose et al., 1998). Findings in our previous study of *F. picrosperma* determined that the species is pollen limited (Grant et al., 2017). Pollen transfer in *F. picrosperma* is predominantly confined to neighboring trees and larger male trees, with more intense floral displays have greater reproductive success than smaller males with less flowers (Grant et al., 2019). Thus, pollinators preferentially travel short distances between conspecific trees likely due to the opportunistic feeding patterns of small insects and lead to the short pollen dispersal distances that characterizes pollen mediated gene flow in *F. picrosperma*.

## 5 CONCLUSION

From our study, we determined that *F. picrosperma* is likely pollinated by an array of small insects, with thrips and beetles a common part of the flower visitor assemblage. This is congruent with observations from many other woody plants in Australian tropical and subtropical rainforests and in the subcanopy of these communities elsewhere where many plant species are pollinated by small, generalist insects. The rainforest subcanopy environment is a distinctive microclimate for pollinators. In this low light, and therefore low energy environment, female flowers of *F. picrosperma* exhibit cost‐saving strategies by not producing nectar and mimicking the smell of reward‐offering male flowers. Male and female flowers displayed the same pattern of floral scent emission, characterized by a decrease in the amount of scent compounds produced as the flower aged. Scent therefore is likely to regulate pollinator behavior and be an important attractant to the species. The main scent constituents found in *F. picrosperma* are ubiquitous in plant floral bouquets and likely makes them indiscriminate to specific taxonomic groups. Our evidence indicates that pollination likely occurs from small insects with low energy requirements that opportunistically feed on or inhabit *F. picrosperma* flowers that offer poor rewards in the shaded subcanopy of the tropical rainforest.

This study improves our understanding of fine‐scale ecological interactions within a poorly studied tropical rainforest community, the AWT, and contributes to the literature of flower visitors to subcanopy tree species which is an area under‐represented compared to canopy tree species. Our focus on ecological aspects of *F. picrosperma* will also help to secure sustainable seed production for commercial manufacture of tigilanol tiglate. Pollinators are of critical importance in natural ecosystems (Garibaldi et al., 2013). Intact forests act as refugia and increase the presence of native pollinators by allowing them to complete their life cycle in a suitable habitat (Ricketts, 2004; Blanche & Cunningham, 2005; Garibaldi et al., 2011). Appreciating the value of the ecosystem services that wild pollinators provide to maintain biodiversity and to enhance fruit and seed production in agricultural crops reinforces the conservation value of remnant tropical rainforest vegetation in the heterogenic landscape where *F. picrosperma* persists.

## CONFLICT OF INTEREST

EcoBiotics Ltd partly funded this research. S.M.O. is a director and shareholder of QBiotics Group Ltd. P.W.R. is a director, employee, and shareholder of EcoBiotics Ltd and QBiotics Group Ltd. The remaining authors declare that they have no conflict of interest.

## AUTHOR CONTRIBUTIONS

**Elektra L. Grant:** Conceptualization (equal); Data curation (lead); Formal analysis (lead); Investigation (lead); Methodology (equal); Project administration (supporting); Validation (lead); Writing‐original draft (lead); Writing‐review & editing (lead). **Helen M. Wallace:** Conceptualization (supporting); Data curation (supporting); Formal analysis (supporting); Investigation (equal); Methodology (equal); Supervision (equal); Validation (equal); Writing‐review & editing (equal). **Peter Brooks:** Data curation (supporting); Methodology (supporting); Resources (supporting); Supervision (supporting); Writing‐review & editing (supporting). **Chris Burwell:** Data curation (supporting); Resources (supporting); Validation (supporting); Writing‐review & editing (supporting). **Paul W. Reddell:** Conceptualization (equal); Funding acquisition (equal); Project administration (supporting); Resources (supporting); Writing‐review & editing (supporting). **Steven M. Ogbourne:** Conceptualization (equal); Funding acquisition (equal); Investigation (supporting); Methodology (supporting); Project administration (lead); Resources (equal); Supervision (lead); Writing‐review & editing (equal).

## Data Availability

The data used in this study will be openly available in the Dryad Digital Repository at https://doi.org/10.5061/dryad.2ngf1vhnn.
